# Comparative Transcriptome Analysis of *Shewanella putrefaciens* WS13 Biofilms Under Cold Stress

**DOI:** 10.3389/fcimb.2022.851521

**Published:** 2022-06-22

**Authors:** Jun Yan, Zhijun Yang, Jing Xie

**Affiliations:** ^1^ College of Food Science & Technology, Shanghai Ocean University, Shanghai, China; ^2^ Laboratory for Quality and Safety Risk Assessment of Aquatic Products in Storage and Preservation of Ministry of Agriculture and Rural Affairs, Shanghai Ocean University, Shanghai, China; ^3^ Shanghai Professional Technology Service Platform on Cold Chain Equipment Performance and Energy Saving Evaluation, Shanghai Ocean University, Shanghai, China; ^4^ National Experimental Teaching Demonstration Center for Food Science and Engineering, Shanghai Ocean University, Shanghai, China

**Keywords:** *Shewanella putrefaciens* WS 13, biofilm, cold stress, transcriptome, specific spoilage organism

## Abstract

*Shewanella putrefaciens* is a Gram-negative bacterium that can cause seafood spoilage under low-temperature conditions. The bacterium easily forms biofilms to enhance its survival in challenging environments. Our previous research revealed that the biofilm formed by *S. putrefaciens* WS13 under the low temperature (4 °C) has larger biomass and tighter structure than at an optimum growth temperature (30 °C). In this study, comparative transcriptome analysis was further performed to get insights into the global-level of gene expression in the biofilm formed by *S. putrefaciens* WS13 under the refrigerating and optimal temperatures using Illumina RNA-Sequencing technique. The results revealed that a total of 761 genes were differentially expressed, of which 497 were significantly up-regulated and 264 were significantly down-regulated (*p*<0.05). The qRT-PCR results of randomly selected differentially expressed genes (DEGs) confirmed the RNA sequencing results. Comparison of transcriptome data revealed 28 significantly changed metabolic pathways under the cold stress, including the down-regulated chemotaxis, and motility, and up-regulated tryptophan metabolism, histidine biosynthesis, and quorum sensing, which benefited the biofilm formation of *S. putrefaciens* WS13 under the adverse circumstance. This study provided useful data for better understanding of the biofilm formation of *S. putrefaciens*, and also laid a theoretical foundation for novel vaccine and drug targets against the severe spoilage bacterium under the cold stress.

## Introduction

*Shewanella putrefaciens* is a severe spoilage bacterium in seafood, particularly under low temperature conditions. The bacterium inhabits ubiquitously in the environment because of its excellent environmental adaptability (Xie et al., 2018; Zhen-Quan et al., 2018). The amount of *S. putrefaciens* cells has often been used as an index to evaluate the quality of seafood (Gram et al., 2002). *S. putrefaciens* can reduce trimethylamine oxide, the umami taste substance in seafood, to trimethylamine, and generate histamine and other harmful volatile substances (Vogel et al., 2005), which poses a serious threat to the seafood processing industry (Xie et al., 2011; Hou et al., 2013).

*S. putrefaciens* is easy to adhere to the surface of food processing equipment such as stainless steel to form a biofilm that is composed of polysaccharides, proteins, nucleic acids, and lipids (Bagge et al., 2001; Flemming and Wingender, 2010). Biofilm can enhance the stress tolerance of bacterial cells (Bagge et al., 2001; Flemming et al., 2016; Yan and Xie, 2021). Recently, our prior study revealed that the biofilm formed by *S. putrefaciens* under 4 °C had 1.61-fold larger biomass and tighter structure than that at 30°C (Yan and Xie, 2020). Therefore, it was speculated that the formation of biofilm could enhance the survival ability of *S. putrefaciens* under the cold stress. However, few information on transcriptome profiles and regulatory factors of *S. putrefaciens* biofilm cells under the cold stress is available so far.

With increased breakthrough of sequeneing technology, RNA-Sequencing (RNA-Seq) technique has been use to study bacterial differential express genes (DEGs), transcript structures, new transcripts and isomers, and alternative splicing and allele-specific expression under adverse circumstances, such as *Clostridium acetobutylicum* in a high-salt environment (Ao et al., 2020), and *Escherichia coli* under different heating methods. In recent years, gene expression between biofilm cells and planktonic cells under stress conditions (Charlebois et al., 2016; Ao et al., 2020), has been investigated at the transcriptomic level in *Clostridium acetobutylicum* (Dong et al., 2015), *Porphyromonas gingivalis* (Lo et al., 2009), and *Gardnerella vaginalis* (Castro et al., 2017). However, current literature on the molecular mechanism of biofilm formation of spoilage bacteria under the cold stress is still rare. Therefore, in this study, we aimed to determine DEGs during the formation of *S. putrefaciens* WS13 biofilm under the cold stress by transcriptomics analysis. The results in this study will provide crucial clues for the targeted inhibition of the biofilm of *S. putrefaciens* under the low temperature.

## Materials and Methods

### Bacterial Strain and Growth Conditions

*S. putrefaciens* WS13 strain was isolated from spoilage shrimp *Litopenaeus vanname* in refrigerator (Chen et al., 2019). The isolate was maintained in Luria Broth (LB, Land Bridge Technology, Beijing, China) with 50% (v/v) glycerol at -80 °C freezer in our laboratory at Shanghai Ocean University, Shanghai, China. *S. putrefaciens* WS 13 was inoculated in 9 mL LB medium (pH 7.4) and incubated at 30°C with shaking at 200 rpm for 12 h, and repeated twice for further analysis.

### Biofilm Assay

Biofilm assay was performed according to the method described by Yan and Xie (2020). S. *putrefaciens* WS13 were incubated overnight to approximately 8 log colony forming units (CFU) mL^-1^ (OD_600nm_≈0.8), and diluted with fresh LB medium (1:100, v/v). A 1 mL of diluted culture was added to each well of sterile 24-well polystyrene microtiter plates. Each sample was tested in six replicates. The samples were incubated at 4°C, and 30°C statically to form biofilms for 24h, and 84 h, respectively. Plastic wraps were used to minimize evaporative loss (Yan and Xie, 2020).

### RNA Extraction, Library Preparation, and RNA Sequencing

Mature biofilm cells of S. *putrefaciens* WS13 at 4°C and 30°C were harvested at 24h, and 84h, respectively. RNA extraction, cDNA library preparation, and RNA sequencing were carried out as described previously (Ao et al., 2020).

### Quality Control and Mapping

Raw paired end reads were trimmed using Fastp v 0.20.0 software (https://github.com/OpenGene/fastp), and low-quality reads and removing reads with size inferior to 50 bp. The clean reads were separately aligned to the reference genome of *S. putrefaciens* WS 13 (GenBank: CP028435.1) with orientation mode using HISAT2 v 2.1.0 software (http://daehwankimlab.github.io/hisat2/). Next, the mapped reads of each sample were assembled using StringTie v 1.3.6 software in a reference-based approach. All sequences were quantified as Fragments Per Kilobase Million Mapped Reads (FPKM) by StringTie. The formula was defined as FPKM = 10^6^×F/(NL×10^-3^), where F is the number of fragments assigned to a certain gene in a certain sample, N is the total number of mapped reads in the certain sample, and L is the length of the certain gene.

### Differential Expression Gene Analysis

DEGs were determined by DEseq2, and genes with FDR <0.05 and |log2 fold change| >1 were identified as DEGs. DEGs were aligned against Gene Ontology (GO, http://geneontology.org/) and Kyoto Encyclopedia of Genes and Genomes (KEGG) (https://www.genome.jp/kegg/) databases. The R package cluster Profiler (http://bioconductor.org/packages/release/bioc/html/clusterProfiler.html) was used to identify enriched GO terms and KEGG pathways with a cut-off of *P*-value < 0.05. DGEs in biofilm formation of *S. putrefaciens* WS 13 were further analyzed using hierarchical clustering. FDR<0.05 is the standard for screening genes with significant differential expression. The gene expression level of *S. putrefaciens* mature biofilm cells at 4°C was used as a reference, whether a gene was up-regulated or down-regulated was determined by comparing its expression level with that at 4°C.

### Quantitative Real-Time Polymerase Chain Reaction Assay

To validate the transcriptome data, ten DEGs were selected randomly for qRT-PCR assay, and 16S rRNA gene was used as the internal reference (**Table 1**). qRT-PCR was using an ABI Stepone Plus Platform (Thermo, USA). Each gene was analyzed in three biological samples, and three reaction repeats were performed for each biological sample as described previously.

**Table 1:** Genes and primers used in the qRT-PCR assay.

Gene ID	Gene name	Forward	Reverse	Fragment size (bp)	TM
AVV84311.1	*rpsD*	CTAACACACCGTAAATACGACGAA	TAAACTGGAAACTGCACCTGGA	110	60
AVV84994.1		GAGTGGTAATAAGGTTGGCGTC	GGTGTATCTGGGCAAGTAGGGT	250	60
AVV84995.1		GTAACTCACCCATACCGGAAATAA	CCCAAGTCTAAAGCAGACCAAG	130	60
AVV82000.1	*sucA*	TGAAGCGGTTGCTTTTGTGT	ATAGATCTTACGTGGTGTAGGGTGT	181	60
AVV84336.1	*fusA*	GGAAAAACGCCGTAAAGAAAA	GTTGAAAATCAGCCAAAGCAA	107	60
AVV85624.1	*gabD*	TAGATGATGTACAGACCTGTCCCG	ACTTTCCCTACTACCAAATTGCG	405	60
AVV86072.1		TTCTGTTATACCCGCTTTGCTTT	CTGTTTAGTCTGTCACGGTTCTGT	298	60
AVV84898.1	*speC*	AGAAGCCTGCTTGTTGTTTGTGT	GGTTGATCGTATTGGTCATCTATGT	173	60
AVV82314.1	*uspE*	AGCATTATTAACCACGCCATC	AATTCAGCATCTAACTGAGCAGC	497	60
AVV84205.1	*katG*	TCGAGCGTTTTAAATGCTTCG	CATGGTGGTAATACCTCCGTCAC	143	60
16S		CGGTGAATACGTTCYCGG	GGWTACCTTGTTACGACTT	128	60

### Statistical Analyses

All the experiments were conducted in six independent biological replicates. Related data to biofilm formation were tested using Duncan’s multiple range test in SPSS 22.0 software (IBM, New York, USA). All data were reported as mean ± standard deviation. Differences with a *p* value < 0.05 were regarded as statistically significant.

## Results and Discussion

### Determination of Transcriptomes of Biofilm Cells Formed by *S. putrefaciens* WS13

Based on our prior research (Yan and Xie, 2020), the biofilm of *S. putrefaciens* WS13 grown in LB medium (pH=7.2) reached maturity at 24 h and 84 h at 4°C and 30°C, respectively (Figures not shown). The cells of mature biofilm at both temperatures were collected, and transcriptomes at a global gene expression level were obtained using Illumina RNA sequencing technique for the further analysis.

### Identification of DEGs in *S. putrefaciens* WS13 Induced by the Cold Stress

**Figure 1:**
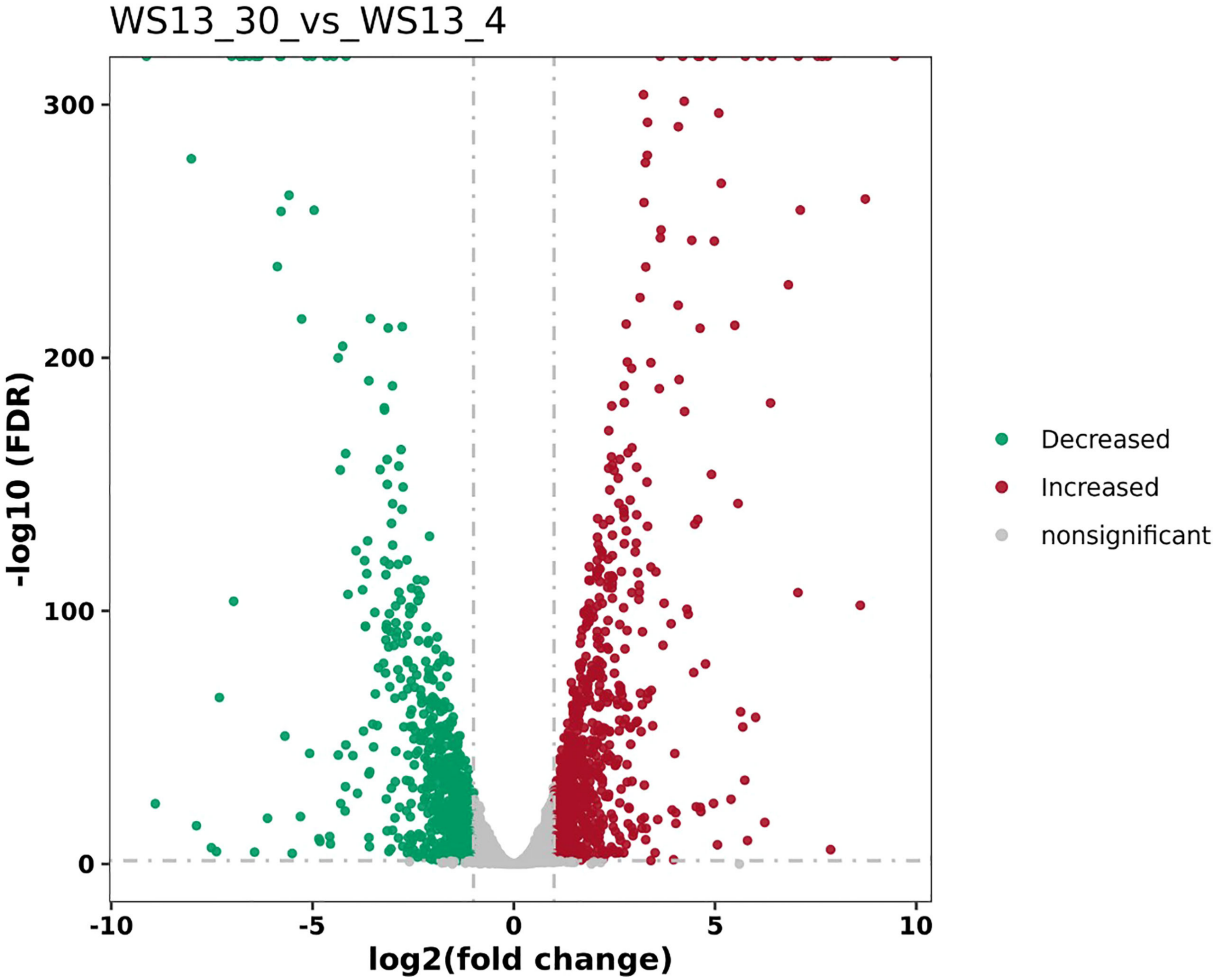
The volcano plots of DEGs between biofilms formed by *S. putrefaciens* WS13 under 4°C and 30°C.

DEGs of the biofilm cells formed by *S. putrefaciens* WS13 at 4°C and 30°C were identified, and the results are shown in **Figure 1**. A total of 761 DEGs were discovered, among which the expression of 497 DEGs was significantly up-regulated, and 264 DEGs were significantly down-regulated (*p <*0.05).

**Figure 2:**
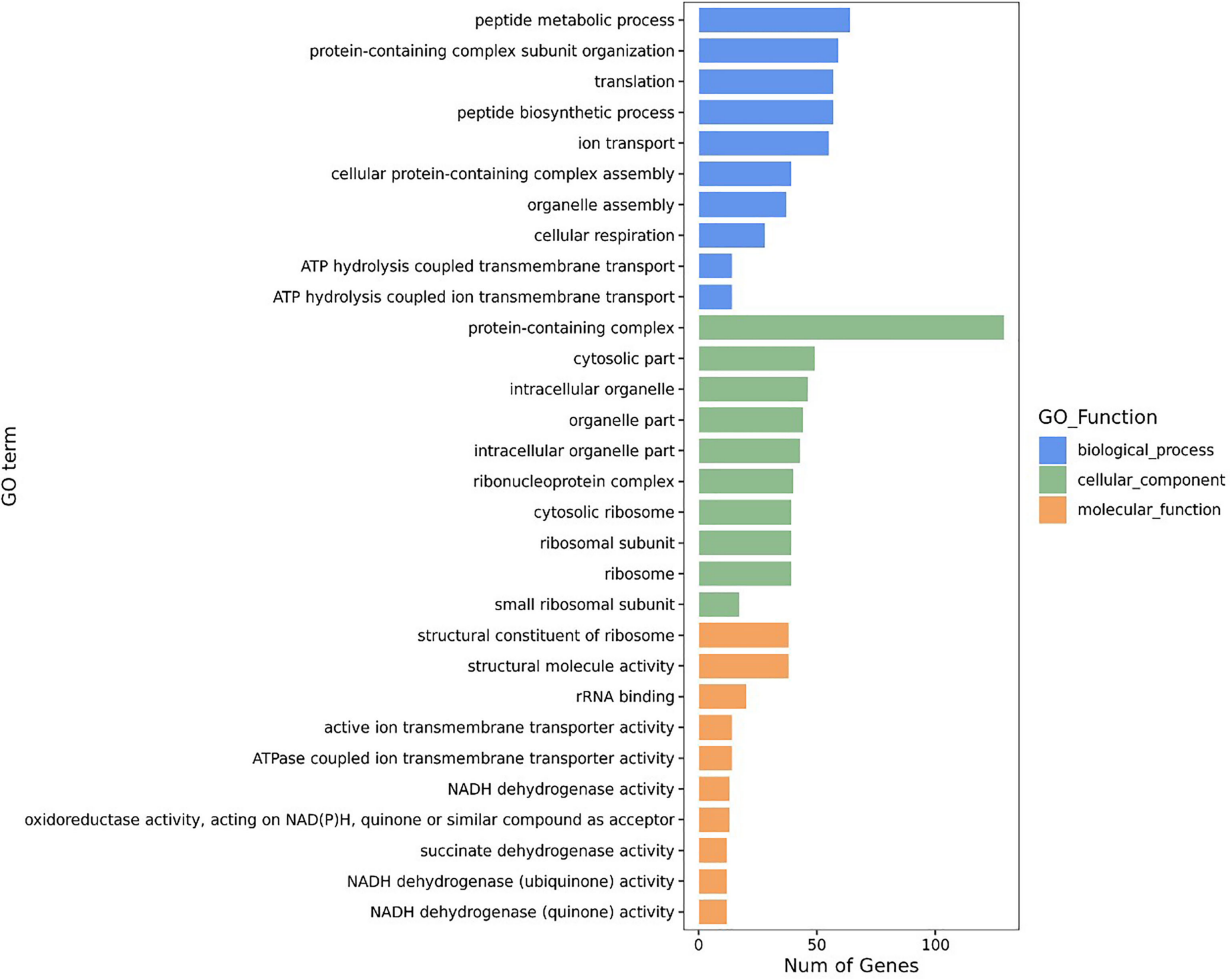
GO enrichment analyses of DEGs.

All DEGs were classified into three major functional categories in the Gene Ontology (GO) database, including biological process (BP), cellular component (CC), and molecular function (MF). The GO enrichment analysis of the DEGs revealed that the most abundant GO function was the peptide metabolic process (11.51%, 64/556), followed by protein-containing complex subunit organization (10.61%, 59/556), translation (10.25%, 57/556), peptide biosynthetic process (10.25%, 57/556), and ion transport (9.89%, 55/556) in BP, whereas cellular respiration (5.04%, 28/556), ATP hydrolysis coupled transmembrane transport (2.52%, 14/556), and ATP hydrolysis coupled ion transmembrane transport (2.52%, 14/556) showed an opposite pattern (**Figure 2**). The protein-containing complex (23.20%, 129/556) was the most enriched DEGs in CC, while the percentages of the DEGs in the structural constituent of ribosome (6.83%, 38/556), and structural molecule activity (6.83%, 38/556) was the highest, followed by the rRNA binding (3.60%, 20/556) in MF (**Figure 2**).

**Figure 3:**
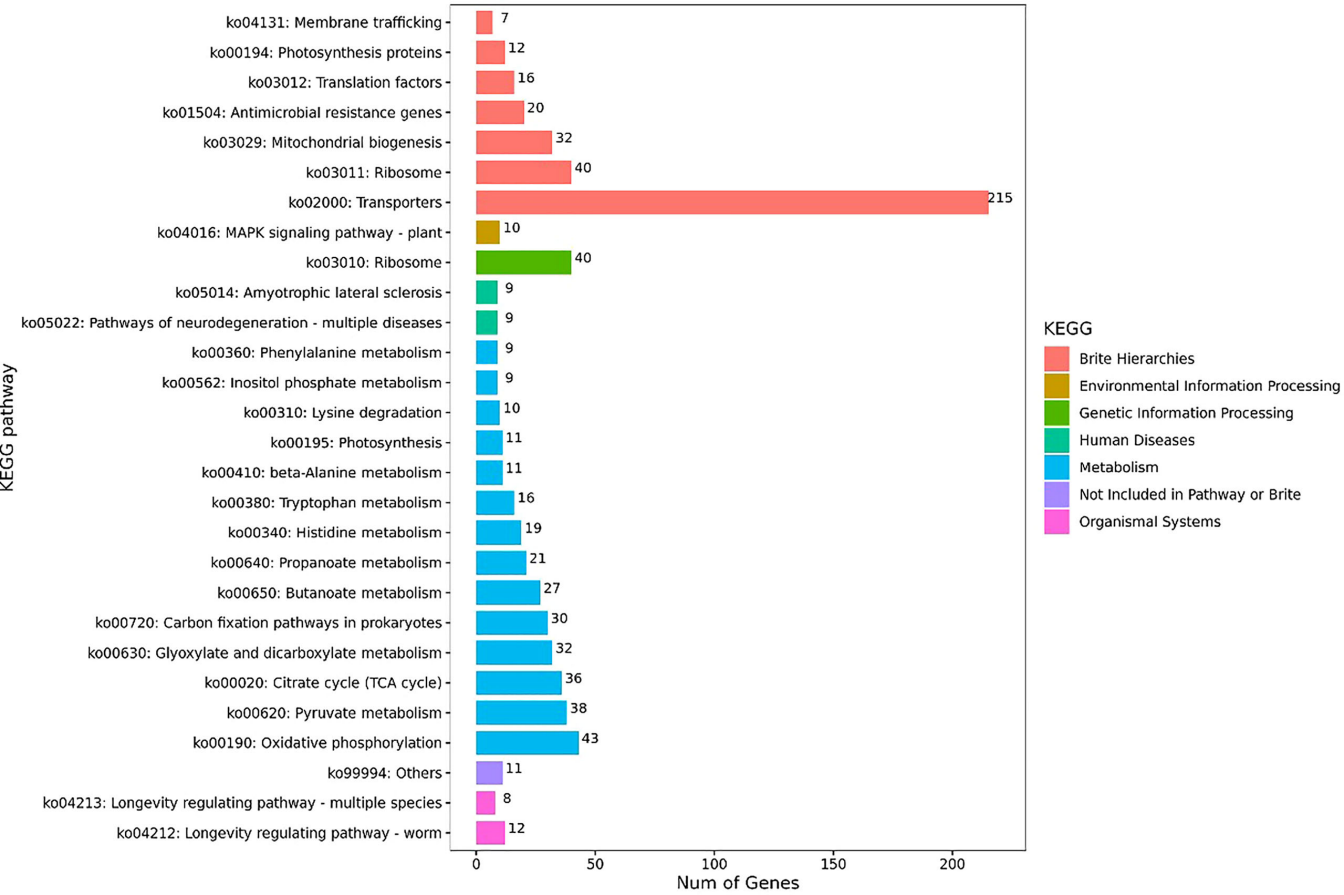
KEGG enrichment analysis of DEGs.

The KEGG pathway enrichment analysis was further performed on the identified DEGs in the obtained transcriptomes of *S. putrefaciens* biofilm cells, and the results revealed 28 significantly changed metabolic pathways, including ribosome, oxidative phosphorylation, citrate cycle, transporters, photosynthesis proteins, histidine metabolism, photosynthesis, tryptophan metabolism, pyruvate metabolism, glyoxylate and dicarboxylate metabolism, carbon fixation pathways in prokaryotes, lysine degradation, MAPK signaling pathway-plants, propanoate metabolism, butanoate metabolism, mitochondrial biogenesis, inositol phosphate metabolism, β-alanine metabolism, longevity regulating pathway-worm, amyotrophic lateral sclerosis, pathways of neurodegeneration-multiple diseases, translation factor, and others, antimicrobial resistance genes, membrane trafficking, phenylalanine metabolism, and longevity regulating pathway-multiple species (**Figure 3**).

### Identified DEGs Involved in Biofilm Formation of *S. putrefaciens* WS13 at the Cold Stress

Four significantly altered metabolic pathways associated with biofilm formation were found in *S. putrefaciens* WS13 at 4°C, including the biofilm formation, amino acid metabolism, two-component system (TCS), and quorum sensing (QS **Table 2**).

**Table 2:** The major DEGs in biofilm cells of *S. putrefaciens* WS13 induced by the cold stress.

Matabolic pathway	Gene ID	Name	Log_2_FC	Definition
Biofilm formation - Pseudomonas aeruginosa				
	AVV85673.1		-2.77	Hypothetical protein SPWS13_3995
	AVV82533.1		-3.75	Crp/Fnr family transcriptional regulator
	AVV82532.1		-5.68	Crp/Fnr family transcriptional regulator
	AVV85155.1	*nrfG*	-2.66	Nitrite reductase
	AVV83548.1		-1.96	GntR family transcriptional regulator
	AVV85962.1	*flgM*	-1.01	Anti-sigma-28 factor FlgM
	AVV85989.1	*flrA*	-1.01	Fis family transcriptional regulator
	AVV85720.1		-1.10	Membrane protein
	AVV85731.1		-1.87	Serine/threonine protein phosphatase
Tryptophan metabolism				
	AVV82001.1	*sucB*	2.79	Dihydrolipoamide succinyltransferase
	AVV85789.1	*amiE*	1.60	Amidase
	AVV82915.1	*katE*	2.84	Catalase
	AVV84271.1		5.68	Aldehyde dehydrogenase
	AVV82993.1		1.69	Enoyl-CoA hydratase
	AVV85788.1	*amiE*	2.36	Amidase
	AVV85097.1	*pdhD*	1.53	Dihydrolipoamide dehydrogenase
	AVV86128.1	*fadJ*	2.12	Multifunctional fatty acid oxidation complex subunit alpha
	AVV82394.1	*bkdB*	1.15	Dihydrolipoamide acetyltransferase
	AVV82994.1		1.17	Enoyl-CoA hydratase
Histidine metabolism				
	AVV82365.1		-3.60	TonB-dependent receptor
	AVV82366.1		-2.85	TonB-dependent receptor
	AVV82367.1		-1.83	TonB-dependent receptor
	AVV84622.1	*hutI*	-2.21	Imidazolonepropionase
	AVV84105.1		-1.24	Arginase
	AVV84398.1	*urdA*	-1.38	Cytochrome C
	AVV82368.1		-1.34	TonB-dependent receptor
	AVV84082.1		-1.21	Hypothetical protein SPWS13_2316
	AVV86163.1	*hutH*	-1.08	Histidine ammonia-lyase
	AVV82091.1	*hisC*	2.88	Histidinol-phosphate aminotransferase
	AVV84271.1		5.68	Aldehyde dehydrogenase
	AVV82088.1		2.89	Hypothetical protein SPWS13_0228
	AVV82089.1	*hisH*	2.58	Imidazole glycerol phosphate synthase
	AVV82090.1	*hisB*	2.52	Imidazoleglycerol-phosphate dehydratase
	AVV82092.1	*hisD*	1.84	Histidinol dehydrogenase
	AVV82087.1	*hisF*	2.35	Imidazole glycerol phosphate synthase
	AVV82086.1	*hisI*	1.94	Phosphoribosyl-AMP cyclohydrolase
	AVV84178.1	*hutH*	1.00	Histidine ammonia-lyase
	AVV82085.1	*hisI*	1.83	Phosphoribosyl-ATP pyrophosphatase
Lysine degradation				
	AVV82001.1	*sucB*	2.79	Dihydrolipoamide succinyltransferase
	AVV84271.1		5.68	Aldehyde dehydrogenase
	AVV82993.1		1.69	Enoyl-CoA hydratase
	AVV85097.1	*pdhD*	1.53	Dihydrolipoamide dehydrogenase
	AVV86128.1	*fadJ*	2.12	Multifunctional fatty acid oxidation complex subunit alpha
	AVV82394.1	*bkdB*	1.15	Dihydrolipoamide acetyltransferase
	AVV82994.1		1.17	Enoyl-CoA hydratase
Two-component system				
	AVV84741.1	*nrfC*	-6.34	4Fe-4S ferredoxin 4Fe-4S ferredoxin
	AVV85250.1	*petA*	-9.12	Ubiquinol-cytochrome C reductase
	AVV83964.1		-3.21	Chemotaxis protein
	AVV82196.1	*hydA*	-3.63	Quinone-reactive Ni/Fe-hydrogenase small chain
	AVV82533.1		-3.75	Crp/Fnr family transcriptional regulator
	AVV84073.1		-2.62	Histidine kinase
	AVV83344.1	*glnA*	-2.11	Glutamine synthetase
	AVV82195.1	*hydB*	-3.36	Hydrogenase 2 large subunit
	AVV85596.1		-2.04	Chemotaxis protein
	AVV83291.1		-1.60	Sensor histidine kinase
	AVV83573.1	*frdA*	-1.82	Fumarate reductase
	AVV86289.1	*cheV*	-1.50	Chemotaxis protein CheW
	AVV85217.1	*CheC*	-1.57	Chemotaxis protein CheC
	AVV83574.1	*frdA*	-1.66	Fumarate reductase
	AVV82532.1		-5.68	Crp/Fnr family transcriptional regulator
	AVV85476.1	*norR*	-1.52	Transcriptional regulator
	AVV84252.1	*glnL*	-1.94	Nitrogen regulation protein NR(II)
	AVV85586.1		-1.79	Amino acid ABC transporter substrate-binding protein
	AVV82193.1	*hyaC*	-2.37	Hydrogenase Ni/Fe-hydrogenase, b-type cytochrome subunit
	AVV82194.1	*hydB*	-2.43	Hydrogenase 2 large subunit
	AVV84209.1	*glnA*	-1.56	Glutamine synthetase
	AVV83483.1		-1.28	Chemotaxis protein
	AVV84098.1		-1.32	Chemotaxis protein
	AVV83827.1		-1.14	Peptidase
	AVV84924.1		-1.35	Cell division protein ZapB
	AVV83129.1	*motA*	-1.45	Flagellar motor protein PomA
	AVV85069.1	*frdC*	-1.56	Fumarate reductase
	AVV82265.1	*ampC*	-1.20	Beta-lactamase
	AVV85068.1	*frdC*	-1.15	Fumarate reductase
	AVV82166.1		-1.00	Chemotaxis protein
	AVV84289.1		-1.85	Histidine kinase
	AVV84253.1	*glnL*	-1.47	Nitrogen regulation protein NR(II)
	AVV84398.1	*urdA*	-1.38	Cytochrome C
	AVV83971.1	*psrB*	-1.96	Polysulfide reductase subunit B
	AVV85962.1	*flgM*	-1.01	Anti-sigma-28 factor FlgM
	AVV84053.1	*maeB*	-1.05	Malate dehydrogenase
	AVV84712.1	*cusB*	-2.01	RND transporter MFP subunit
	AVV84254.1	*glnL*	-1.32	Nitrogen regulation protein NR(II)
	AVV84075.1		-1.54	Response regulator receiver protein
	AVV83905.1		-1.15	Chemotaxis protein
	AVV85989.1	*flrA*	-1.01	Fis family transcriptional regulator
	AVV85931.1		-1.09	Membrane protein cyd operon protein YbgT
	AVV83697.1	*glnK*	-1.66	Nitrogen regulatory protein P-II 1
	AVV85720.1		-1.10	Membrane protein
	AVV85990.1	*flrB*	-1.38	Sensor histidine kinase
	AVV83549.1	*cydA*	-2.29	Cytochrome D ubiquinol oxidase subunit I
	AVV85070.1	*frdA*	-1.23	Fumarate reductase
	AVV84287.1	*frdC*	-4.57	Fumarate reductase
	AVV83377.1	*glnB*	-1.11	Nitrogen regulatory protein P-II
	AVV84286.1	*frdB*	-4.81	Fumarate reductase iron-sulfur subunit
	AVV83550.1	*cydB*	-1.80	Ubiquinol oxidase subunit II, cyanide insensitive
	AVV83918.1	*ttrB*	-1.97	Tetrathionate reductase subunit B
	AVV84284.1	*frdA*	-4.55	Fumarate reductase flavoprotein subunit
	AVV86161.1		-2.76	Cytochrome C flavocytochrome c
	AVV85991.1	*flrC*	-1.11	Fis family transcriptional regulator
	AVV84288.1	*frdD*	-3.58	Fumarate reductase
	AVV84265.1	*cpxA*	-1.06	Sensor histidine kinase
	AVV83919.1	*ttrC*	-1.95	Polysulfide reductase
	AVV84285.1	*frdA*	-1.65	Fumarate reductase flavoprotein subunit
Quorum sensing				
	AVV84313.1	*secY*	2.27	Preprotein translocase subunit SecY
	AVV86104.1	*secF*	2.06	Preprotein translocase subunit SecF
	AVV84314.1	*secY*	2.13	Preprotein translocase, SecY subunit
	AVV84540.1	*yidC*	1.61	Membrane protein insertase
	AVV85368.1		2.07	RND transporter
	AVV84996.1		1.41	Outer membrane adhesin-like protein
	AVV83999.1		1.41	Hypothetical protein SPWS13_2218
	AVV86108.1		4.64	Peptidase S8
	AVV82086.1	*hisI*	1.94	Phosphoribosyl-AMP cyclohydrolase
	AVV82884.1	*dppB*	1.22	Peptide ABC transporter permease
	AVV82009.1		1.72	Hypothetical protein SPWS13_0149
	AVV86102.1	*yajC*	1.14	Preprotein translocase subunit YajC
	AVV86191.1	*trpG*	1.13	Anthranilate synthase subunit II
	AVV82085.1	*hisI*	1.83	Phosphoribosyl-ATP pyrophosphatase
	AVV84384.1		1.11	Cytochrome B561
	AVV82919.1	*gadB*	1.76	Glutamate decarboxylase
**Carbohydrate metabolism**				
	AVV83139.1	*ykgG*	-3.15	L-lactate dehydrogenase complex protein LldG
	AVV83137.1	*Nan*	-3.32	L-lactate dehydrogenase complex protein LldE
	AVV83138.1	*Nan*	-3.01	L-lactate dehydrogenase complex protein LldF
	AVV85349.1	*Nan*	-1.53	Acetoin utilization protein AcuB
	AVV85417.1	*sfsA*	-1.26	Sugar fermentation stimulation protein A
**Energy metabolism**				
	AVV84740.1	*nrfB*	-6.79	Cytochrome c nitrite reductase small subunit
	AVV84741.1	*nrfC*	-6.35	Polysulfide reductase chain B
	AVV84742.1	*nrfD*	-5.78	Protein NrfD
	AVV85151.1	*ccmF*	-3.50	Cytochrome c-type biogenesis protein NrfE
	AVV85149.1	*ccmH*	-3.58	Formate-dependent nitrite reductase complex subunit NrfG
	AVV85155.1	*nrfG*	-2.67	Formate-dependent nitrite reductase complex subunit NrfG
	AVV83971.1	*psrB*	-1.96	Polysulfide reductase chain B
	AVV82007.1	*Nan*	-1.06	Ferredoxin/flavodoxin—NADP+ reductase
	AVV83970.1	*psrC*	-1.72	Polysulfide reductase chain C
	AVV85150.1	*ccmF*	-3.15	Cytochrome c-type biogenesis protein NrfE

Some genes encoding transcripitional regulators were slightly down-regulated (*p*<0.05), such as *flgM*, and *flrA* genes. The former that encodes an anti-sigma-28 factor FlgM can regulate the expression of flagellar genes in a complex regulatory network controlling chemotaxis, swimming and biofilm formation in *Rhodobacter sphaeroides* (Wilkinson et al., 2011). It has been reported that the *flrA* gene that encodes Fis family transcriptional regulator was strongly sensitive to environmental stress (Gang et al., 2016). The silencing of the *flrA* gene led to deficiencies in adhesion, motility, flagellar assembly, biofilm formation and exopolysaccharide (EPS) production in *Vibrio alginolyticus* (Gang et al., 2016). These results suggested that the reduction of flagellar synthesis and motility may have enhanced the biofilm formation of *S. putrefaciens* WS13 at 4°C.

### Identified DEGs Involved in Amino Acid Metabolism in *S. putrefaciens* Biofilm at the Cold Stress

The biofilm is composed of extracellular substances secreted by *S. putrefaciens* WS13, such as polysaccharides, proteins, lipids, and other substances. Proteins play a crucial role in maintaining the structural stability of biofilms (Yan and Xie, 2020). Amino acids are key intermediates in both carbon and nitrogen metabolisms in microorganisms. Bacterial amino acid metabolism is usually sensitive to environmental stress. In this study, comparative transtriptomics analyses revealed remarkedly changed DEGs in the amino acid metabolism in biofilm cells of *S. putrefaciens* WS13 under the cold stress. These DEGs were significantly enriched in the amide biosynthetic process, peptide metabolic process, translation, peptide biosynthetic process, and other protein-related GO functions. For example, interestingly, all the DEGs involved in the tryptophan metabolism were significantly up-regulated in the *S. putrefaciens* WS13 biofilm at 30°C, including the *sucB, amiE, katE, pdhD, fadJ*, and *bkdB* genes (2.23 fold to 7.15 fold) *(p<0.05)*. It has been reported that the tryptophan biosynthesis genes were up-regulated in the biofilms of *Escherichia coli* and *Salmonella enterica* (Domka et al., 2007; Hamilton et al., 2009). It was shown that exogenous tryptophan significantly accelerated the biofilm formation of *S. enterica* and *Fusobacterium nucleatum* and completely restored the deleted mutant of *S. enterica* in biofilm formation (Hamilton et al., 2009; Sasaki-Imamura et al., 2010). In the amino acid metabolism, some DEGs involved in histidine metabolism were significantly up-regulated in *S. putrefaciens* WS13 at 4°C (2.23 fold to 61.60 fold) (*p*<0.05). It has been reported that under the acetate, butyrate, or butanol stress, the genes involved in histidine biosynthesis were up-regulated in *C. acetobutylicum* (Alsaker et al., 2010; Wang et al., 2013). The induction of histidine biosynthesis genes was also observed under the acid tolerance in *Lactobacillus casei* (Broadbent et al., 2010). Amino acid metabolism played a vital role in bacterial adaptation to certain circumstances including metabolite stress (Alsaker et al., 2010; Wang et al., 2013), oxygen tolerance (Hillmann et al., 2009), and sporulation (Jones et al., 2008). The highly increased amino acid metabolism may enable bacterial cells to adjust the structure and function of biofilm in response to the cold stress.

### Identified DEGs Involved in TCSs in *S. putrefaciens* Biofilm at the Cold Stress

Bacteria have a variety of signal transduction systems, which can sense external signal stimuli and respond adaptively to changes (e.g., osmolarity, light, temperature, and oxygen) in the surrounding environment (Liu et al., 2013). TCS that compose of histidine protein kinases (HKs) and response regulators (RRs) are widely present in gram-negative bacteria. TCSs are very important signaling pathways that coordinate responses to environmental stimulus, and regulates bacterial sporulation, biofilm formation, competence, and chemotaxis (Sieuwerts et al., 2010). In this study, comparative transcriptomic analyses revealed 59 DEGs involved in the TCSs, which were all significantly up-regulated in *S. putrefaciens* WS13 at 4°C, e.g., *cheV* (2.84 fold), *motA* (2.75 fold). For example, bacterial chemotaxis system is a typical coupling protein-dependent signal transduction system and play a crucial role in bacterial colonigetion and adhesion. It has been reported that the up-regulated genes involved in chemotaxis significantly reduced the ability of adhesion of the organism, motility, chemotaxis, and biofilm formation in *Vibrio harveyi*. Environmenal factors such as temperatures, salinities, and pH values affected the chemotactic gene expression involved in the regulation of adhesion ability (Xu et al., 2021). In this study, the *cheW* and *cheC* genes were significantly down-regulated in the biofilm formation by *S. putrefaciens* at the cold stress. The *cheV* gene encodes a linker protein, while the *cheC* encoding protein has phosphatase activity (Moon et al., 2016). Moreover, expression of six additional genes coding for chemotaxis proteins were all reduced at the transcriptional level in *S. putrefaciens* at 4°C, including the AVV85596.1 (0.24 fold), AVV83483.1 (0.41 fold), AVV84098.1 (0.40 fold), and AVV83905.1 (0.45 fold). These results suggested inhibital chemotaxis and/or motility of the bacterium. The movement of flagella *via* the flagellar motor complex affected mature biofilm architecture (Wood et al., 2006). In this study, the motA gene that encodes a key component of the motor compelx was significantly down-regulated (-1.45 fold) (*p*<0.05) suggesting poor motility forming flatter microcolony structures of *S. putrefaciens* biofilm at 4°C. Additionally, the DEGs enriched in TCSs may sense external signal stimulation and regulate the movement ability of microbial cells, thereby affecting the biofilm formation.

### Identified DEGs Involved in QS in *S. putrefaciens* Biofilm at the Cold Stress

Biofilm formation is essentially coordinated through a cell density-dependent gene regulation system known as QS (Fuqua et al., 1994; Kjelleberg and Molin, 2002; Parsek and Greenberg, 2005). In this study, remarkly, the DEGs involved in the QS were significantly down-regulated in *S. putrefaciens* at 4°C, including *secY* (4.83 fold), *secF* (4.20 fold), *hisI* (3.83 fold), *gadB* (3.38 fold), *yidC* (3.06 fold), *dppB* (2.34 fold), *yajC* (2.21 fold), *trpG* (2.19 fold). For example, YidC has been recognized as a drug to inhibit biofilm formation in *Staphylococcus aureus* (Hofbauer et al., 2018). Expression of the *yidC* gene was significantly influenced bu pH and starvation stress in *Vibrio alginolyticus*, and the bacterial adhesion was significantly decreased after silencing of the *yidC* gene (Lemos et al., 2004). YidC function as integral membrane chaperone/insertase associated with the classical SecYEG translocon, which could contribute to inhibit biofilm formation (Hofbauer et al., 2018). The upregulated expression of the *secY* and *yidC* genes involved in extracellular polymeric substances was also observed in the stage of biofilm maturation of *Bifidobacterium Longun* FGSZY16M3 (Liu et al., 2021). In this study, the *secF* gene expression was also highly increased by 2.06 fold, which encodes a component of the Sec translocon. Additionally, expression of the *yajC* (1.14 fold), *dppB* (1.22fold), and AVV84996.1 (1.41 fold) were significantly increased in *S. putrefaciens* WS13 biofilm at the cold stress, which encode a potein translocase subunit YajC, a peptide ABC transporter permease, and an outer membrane adhesin-like protein, respectively. These results suggested QS plays an important role in the biofilm formation of *S. putrefaciens* at the cold stress.

### Identified DEGs Involved in the Other Key Metabolic Pathways in *S. putrefaciens* Biofilm at the Cold Stress

Energy metabolism such as carbohydrate metabolism, pyruvate metabolism, oxidative phosphorylation, and citrate cycle helps bacteria adapt to the changing environment. In this study, approximately 5 DEGs involved in the carbohydrate metabolism were significantly down-regulated (0.10 fold to 0.41 fold) in *S. putrefaciens* WS13 biofilm formed at 30°C (*p*<0.05). For energy metabolism, approximately 10 DEGs were significantly down-regulated (0.01 fold to 0.48 fold) at 30°C. For example, expression of the *ppsA* gene was increased by 2.62 fold in *S. putrefaciens* at 4°C which can promote biofilm formation by enhancing bacterial adhesion (Ao et al., 2020). Moreover, approximately 11 DEGs involved in aminoacyl-tRNA biosynthesis were highly down-regulated (2.02 fold to 5.15 fold) in *S. putrefaciens* WS13 biofilm at 4°C, which may have released amino acids to feed the energy-providing pathways (Bénédicte et al., 2009), and benefited the survival of *S. putrefaciens* WS13 under the cold stress.

### Confirmation of the DEGs by the qRT-PCR Assay

The relative expression levels of randomly selected DEGs were determined and calculated using 16S rRNA as the internal reference gene, including: AVV84311.1, AVV84994.1, AVV84995.1, AVV82000.1, AVV84336.1, AVV85624.1, AVV86072.1, AVV84898.1, AVV82314.1, and AVV84205.1. The obtained qRT-PCR results (**Table 3**) confirmed the transcription changes of these DEGs in the comparative transcriptome analyses.

**Table 3:** The results of the qRT-PCR assay.

Index	Sample Name	Assay Name	mean ΔCT	ΔΔCT	Fold Change	Up/Down	Control
1	S30	AVV84311.1	-11.36	-2.87	7.29	Up	16S
	WS4	AVV84311.1	-8.49				
2	S30	AVV84994.1	-8.63	-5.23	37.46	Up	16S
	WS4	AVV84994.1	-3.40				
3	S30	AVV84995.1	-10.56	-3.96	15.52	Up	16S
	WS4	AVV84995.1	-6.60				
4	S30	AVV82000.1	-8.12	-4.33	20.06	Up	16S
	WS4	AVV82000.1	-3.79				
5	S30	AVV84336.1	-8.30	-3.54	11.67	Up	16S
	WS4	AVV84336.1	-4.75				
6	S30	AVV85624.1	-4.72	2.30	0.20	Down	16S
	WS4	AVV85624.1	-7.02				
7	S30	AVV86072.1	-5.59	2.57	0.17	Down	16S
	WS4	AVV86072.1	-8.16				
8	S30	AVV84898.1	-5.43	5.29	0.03	Down	16S
	WS4	AVV84898.1	-10.72				
9	S30	AVV82314.1	-3.06	2.26	0.21	Down	16S
	WS4	AVV82314.1	-5.32				
10	S30	AVV84205.1	-5.54	1.85	0.28	Down	16S
	WS4	AVV84205.1	-7.39				

## Conclusions

This study was the first to characterize the global-level gene expression of biofilm cells of *S. putrefaciens* WS13 under the cold stress. Distinct transcriptomic profiles were obtained using Illumina RNA sequencing technique. Comparative transcriptomic analyses revealed a total of 761 DEGs in the biofilm formed at 4°C, among which the expression of 497 DEGs was significantly up-regulated, and 264 DEGs were significantly inhibited (*p*<0.05). Although carbohydrate and energy metabolisms were repressed in the biofilm cells at the harsh condition, *S. putrefaciens* WS13 reduced chemotaxis, and mobility, but enhanced histidine biosynthesis, tryptophan metabolism, and QS to construct the biofilm and survive at the cold stress. This work provides valuable insights into the transcriptiomic regulation in biofilm cells under cold stress and laid a theoretical foundation for the targeted inhibition of the biofilm formation of the severe spoilage *S. putrefaciens* WS13.

## Data Availability Statement

Raw data of the transcriptomes was deposited in NCBI database under the accession number PRJNA759975 (http://www.ncbi.nlm.nih.gov/bioproject/759975).

## Author Contributions

JX, JY conceived the idea. JY carried out the laboratory work and wrote the paper. ZY assisted in the data analysis. JX revised the manuscript. All authors have read and approved the final manuscript.

## Funding

This work was supported by the National Natural Science Foundation of China (31972142), Shanghai Engineering Research Center Construction Special Fund from Shanghai Municipal Science and Technology Commission (19DZ2284000), the Startup Foundation for Young Teachers of Shanghai Ocean University.

## Conflict of Interest

The authors declare that the research was conducted in the absence of any commercial or financial relationships that could be construed as a potential conflict of interest.

## Publisher’s Note

All claims expressed in this article are solely those of the authors and do not necessarily represent those of their affiliated organizations, or those of the publisher, the editors and the reviewers. Any product that may be evaluated in this article, or claim that may be made by its manufacturer, is not guaranteed or endorsed by the publisher.
